# A Vaccination Difficulty

**Published:** 1907-01-12

**Authors:** 


### A VACCINATION DIFFICULTY.

An important point with regard to re-vaccination has been settled by the Local Government Board. In a certain union a case of small-pox occurred. The guardians, being sensible persons, ordered the public vaccinator to re-vaccinate all who belonged to the family of the sufferer, and also those who lived near him or were supposed to have been in any way exposed to the infection of the disease. Among these were the patient's own children and some others who lived in close proximity to his dwelling. For these re-vaccinations the guardians paid, but the district auditor, when he went over the accounts, disallowed the charge in the case of all children under ten years of age, and surcharged the guardians who had ordered the payment. His reasons for this decision were (1) Because there is no provision, statutory or otherwise, for re-vaccination under the age of ten years, and consequently no fee is payable for such operation; and (2) Because the payment made was not in respect of a debt legally due or recoverable from the guardians. Naturally the guardians involved referred the point to the Local Government Board, who took a common-sense view of the matter. They held that the circumstances were such as to render applicable to the case Section 28 of the Vaccination Act of 1867, which authorises guardians to pay out of their funds all reasonable expenses incurred by them in taking measures to prevent the spread of small-pox. Thus the incident closes in a satisfactory way. It would have been a distinct misfortune if a Board of Guardians so favourably disposed to preventive measures had been discouraged by such a surcharge. Now they and other boards will realise that in the matter of vaccination, where it is proved to be desirable, they have the support of the central authority.

